# Berlin as a Medical Centre

**Published:** 1885-07

**Authors:** 


Berlin as a Medical Centre. A Guide for American Students and Practitioners. By Horatio R. Bigelow, M.D. New England Publishing Co., Sandy Hook, Conn., 1885.

This little book, of about 100 pages, is destined to fill a much-needed want to American medical men desiring to visit Berlin for study, and is not without value to any person proceeding Europewards.

He begins by telling one how to get to Europe, and then carries him to Berlin, recommending hotels, boarding-houses, restaurants, book-sellers, etc., and thereby doing much to save the uninitiated from falling into the hands of the innumerable swindlers that throng any great city. From some six years of life in medical Berlin we can certainly appreciate the value of this book to any one going there. The only objection we have to it is the ridiculously low scale of expenses which the author places as among the possibilities.

Berlin is one of the very dearest cities in the world to live in ; especially is it so to one who cannot eat the ordinary fatty, unsavory German cooking. When the author says a person can go to Berlin, and study for six months, and return to New York, for either $154.09 or $340.00, he labors under a mistake. All we can say is this, that any medical student who desires to go to Berlin, live like a gentleman should, without a cent of extravagance, study hard, dress well, and eat well, must calculate on at least $75.00 per month for living expenses alone, and will find $100.00 a much more convenient sum. Books and instruments cannot be bought on any such allowance.

The author recommends pensions or boarding-houses. They may do to go to until one is acquainted, but far better are any of the second-class hotels recommended by him, taking a room only and eating at pleasure. With due regard to the cautions given about hiring rooms, one can find pleasant rooms and kind attendance more easily in Berlin than any American city.

Except that the book is liable to deceive about expenses, it is an admirable guide to all. B.

